# Postpartum woman with pneumomediastinum and reverse (inverted) takotsubo cardiomyopathy: a case report

**DOI:** 10.1186/1752-1947-8-89

**Published:** 2014-03-05

**Authors:** Sebastian Niko Nagel, Michael Deutschmann, Eric Lopatta, Michael Lichtenauer, Ulf Karl Martin Teichgräber

**Affiliations:** 1 Department of Radiology, Jena University Hospital, Erlanger Allee 101, 07747 Jena, Germany; 2 Department of Internal Medicine I (Cardiology and Intensive Care Medicine), Jena University Hospital, University Heart Center Jena, Erlanger Allee 101, 07747 Jena, Germany

**Keywords:** Inverse takotsubo cardiomyopathy, Postpartum chest pain, Postpartum pneumomediastinum

## Abstract

**Introduction:**

Pneumomediastinum is known to occur during labor. Patients typically present with chest pain and symptoms may be suspicious, for example of pulmonary embolism or aortic dissection. The condition itself, however, is rather harmless and self-limiting.

Takotsubo cardiomyopathy is associated with psychologically or physiologically stressful events and its symptoms mimic myocardial infarction. Yet, symptoms often improve quickly as the initially impaired cardiac function is usually restored within days or weeks.

Although the initial presentation of the patient in this case report was dramatic, the clinical course was positive and the patient could be quickly dismissed in a good general condition. To the best of our knowledge, no presentation of a combined occurrence of postpartum pneumomediastinum and reverse (inverted) takotsubo cardiomyopathy exists.

**Case presentation:**

We present the case of a 30-year-old Caucasian woman with sudden onset of thoracic back and chest pain approximately 24 hours after an otherwise unremarkable vaginal delivery. A contrast-enhanced chest computed tomography showed cervical and mediastinal emphysema without proof for pulmonary embolism or aortic dissection. She received a symptomatic analgesic treatment and was dismissed to the obstetrics department for monitoring.

Within hours, slightly increased levels of troponin I were observed without corresponding electrocardiography changes. Immediate cardiac catheterization and a cardiovascular magnetic resonance imaging (performed within 24 hours) revealed basal to midventricular hypokinesia, but were otherwise unremarkable. A low-dose treatment for congestive heart failure was initiated, under which symptoms subsided within days. She was dismissed after 12 days in a good general condition.

**Conclusions:**

Although the clinical presentation of the combination of the diseases initially was dramatic, the prognosis is positive. In the context of the preceding delivery, knowledge about the postpartum pneumomediastinum lets the radiologist of the emergency department quickly make this diagnosis. The takotsubo cardiomyopathy, however, needs broader diagnostics to not miss intervention-requiring causes.

## Introduction

### Postpartum pneumomediastinum

Pneumomediastinum describes air or other gas in the mediastinum (mediastinal emphysema). The idiopathic form is also referred to as “Hamman’s Syndrome”, named after Louis Hamman who established its clinical features [1]. The postpartum pneumomediastinum is a spontaneous variant associated with labor. As described by Macklin and Macklin [2], straining similar to the Valsalva maneuver leads to an increase of intrathoracic pressure: membranes of marginal alveoli rupture, air escapes into the surrounding tissue and travels via the pulmonary interstitium and hilum into the mediastinum (“Macklin effect”). There is no causative treatment and the emphysema usually is self-limiting [3]. Only in severe cases may decompression of the emphysema become necessary [3]. It is typically associated with retrosternal chest pain radiating to the back [3].

### Takotsubo cardiomyopathy

Synonyms: stress cardiomyopathy, stress-induced cardiomyopathy, apical ballooning cardiomyopathy, transient apical ballooning syndrome, broken heart syndrome.

In this cardiomyopathy an apical ballooning of the left ventricle is observed with hypo- to akinesia of the apical parts and compensatory hyperkinesia of the basal parts [4]. The resulting shape resembles a Japanese octopus trap called “takotsubo”. Synonyms like “stress-induced cardiomyopathy” or “broken heart syndrome” underline its association with psychologically or physiologically stressful events [4]. However, the akinesia does not necessarily need to affect the apex, as case reports with basal to midventricular myocardial dysfunction show [5-7]. Typically, myocardial enzymes are raised and the electrocardiography (ECG) is abnormal. However, cases without ECG changes [8] and normal levels of myocardial enzymes [9] are reported. Symptoms mimic myocardial infarction and usually subside within weeks [4].

The pathophysiology of the takotsubo cardiomyopathy is not understood yet. Common theories suspect a vascular dysfunction, ranging from vasospasms of the coronary arteries [10] to microvascular dysfunctions [11,12]. Patients also showed levels of catecholamines beyond those of patients with myocardial infarction [13], what on the one hand may explain the association of takotsubo cardiomyopathy to stressful events and on the other could be a potential trigger of the cardiomyopathy itself [14]. However, it is beyond the scope of this case report to provide a complete review of the literature.

## Case presentation

We present the case of a 30-year-old Caucasian woman with sudden onset of thoracic back and chest pain approximately 24 hours after an otherwise unremarkable vaginal delivery.

**Figure 1:**
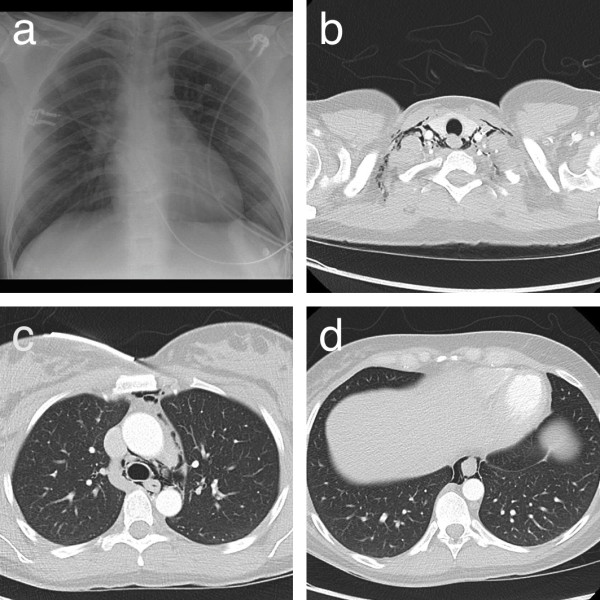
**X-ray and chest computed tomography at initial presentation. a** shows the chest X-ray of the patient that reveals a thin stripe of increased transparency along the left side of her trachea. **b** to **d** show slices of the chest computed tomography that reveal cervical and mediastinal emphysema.

In consideration of the clinical presentation, a contrast-enhanced chest computed tomography (CT) was performed to rule out pulmonary embolism. D-dimer was also elevated (up to 2700μg/L), but since also normal pregnancies cause a progressive increase in blood levels [15], benefit of this parameter was limited. The CT showed cervical and mediastinal emphysema (Figure 1) without proof for pulmonary embolism or aortic dissection. As no reason for her emphysema could be identified, in the context of the preceding delivery her emphysema was interpreted as spontaneous postpartum pneumomediastinum. She received analgesics and was dismissed to the obstetrics department for monitoring. No specific treatment was performed. According to the prescribing information of the contrast agent (Ultravist® 300; Bayer Schering Pharma, Berlin, Germany) nursing was paused for 48 hours.

**Figure 2:**
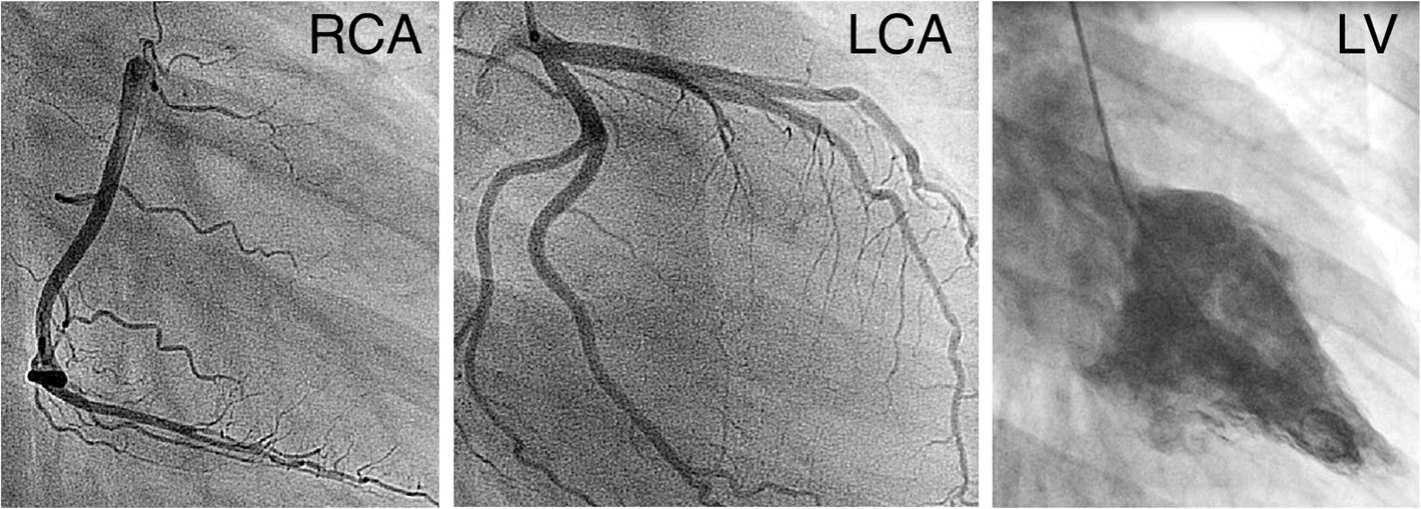
**Cardiac catheterization.** Hemodynamically not relevant kinking of the left anterior descending artery. Coronary arteries are otherwise unremarkable. Left ventricle angiography suggests basal to midventricular ballooning. Abbreviations: LCA, left coronary artery; RCA, right coronary artery; LV, left ventricle angiography.

**Figure 3:**
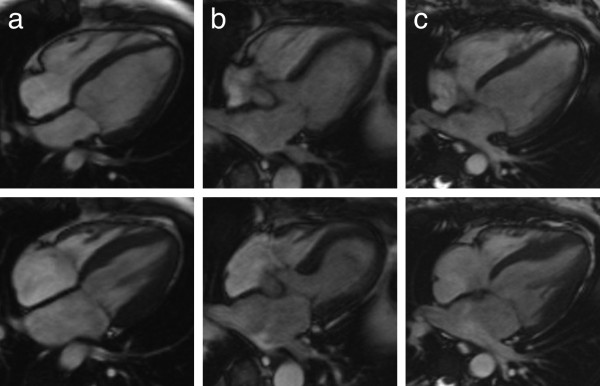
**Native cardiac magnetic resonance imaging cine sequences.** The row above shows the end diastolic, the row below the end systolic state of the cardiac cycle. Column **a** shows a patient with normal left ventricular function and a symmetric contractility of the myocardium throughout the left ventricle, **b** shows a patient with takotsubo cardiomyopathy with normal contractility of the basal myocardium and impaired apical contractility with ballooning, **c** is the patient of this case report with reverse (inverted) takotsubo cardiomyopathy with normal contractility of the apical myocardium and impaired contractility of the basal parts.

Within hours after the CT scan, slightly increased levels of troponin I (up to 1.88ng/mL) were observed. The patient’s ECG showed sinus tachycardia, but no other changes such as ST elevation or T inversion. Her left ventricle had an impaired function (ejection fraction, EF, 19%); echocardiography (Table 1) as well as cardiac catheterization (Figure 2) revealed basal to midventricular hypokinesia, but were otherwise unremarkable. A cardiac magnetic resonance imaging performed within 24 hours after the initial presentation confirmed these findings (Figure 3). A low-dose treatment for congestive heart failure with beta-blockers and angiotensin-converting enzyme inhibitors was initiated, under which symptoms subsided within days. A peripartum cardiomyopathy was also considered, but the patient’s age, the fact that it was her first pregnancy, missing signs of a dilative cardiomyopathy as well as the course of the cardiac symptoms made the takotsubo cardiomyopathy more likely [16]. Also, symptoms and clinical findings fulfilled the diagnosis criteria of the takotsubo cardiomyopathy [17]. However, a therapy with bromocriptine was initialized to prevent prolactin secretion, as positive effects on the course of patients with peripartum cardiomyopathy have been observed [16].

**Table 1:** Course of left ventricular function in echocardiography from day 0 to day 12

**Day**	**EDV (cc)**	**ESV (cc)**	**SV (cc)**	**LVDd (mm)**	**LVDs (mm)**	**EF (TomTec, %)**
0	200	160	40	56	48	19
1	-	-	-	-	-	33
2	140	78	62	-	-	45
5	125	60	65	49	38	53
12	100	40	60	47	27	60

Abbreviations: *EDV* end-diastolic volume, *EF* (TomTec) ejection fraction calculated by TomTec, 4D LV-Analysis© imaging software, Unterschleissheim, Germany, *ESV* end-systolic volume, *LVDd* left ventricular end-diastolic dimension, *LVDs* left ventricular end-systolic dimension, *SV* stroke volume, - unavailable.

After 12 days the patient’s echocardiography showed restored left ventricular function (EF 60%, Table 1) and she was dismissed after a noticeable improvement of her general condition. The ECG remained unremarkable.

## Conclusions

Although the clinical presentation of both diseases initially was dramatic, the prognosis is positive. In the context of the preceding delivery, knowledge about the postpartum pneumomediastinum lets the radiologist of the emergency department quickly make this diagnosis. The takotsubo cardiomyopathy, however, needs broader diagnostics to not miss intervention-requiring causes.

## Consent

Written informed consent was obtained from the patient for publication of this case report and any accompanying images. A copy of the written consent is available for review by the Editor-in-Chief of this journal.

## Abbreviations

CT: Computed tomography; ECG: Electrocardiography; EF: Ejection fraction.

## Competing interests

The authors declare that they have no competing interests.

## Authors’ contributions

SN analyzed and interpreted the patient data. He was the major contributor in writing the manuscript. MD and EL confirmed the radiological image evaluation and proofread the manuscript. ML supplied images of the cardiac catheterization as well as the echocardiography and provided cardiologic knowledge about the patient. UT was involved in writing and proofreading the manuscript. All authors read and approved the final manuscript.
